# The value of p16^INK4a^ immunostaining for high-grade squamous intraepithelial lesions in human papillomavirus-negative patients

**DOI:** 10.1186/s12905-022-01714-0

**Published:** 2022-04-27

**Authors:** Dai Zhang, Jie Song, Xiaosong Zhang, Hui Bi

**Affiliations:** 1 Department of Obstetrics and Gynecology, Peking University First Hospital, No. 8 Xishiku Street, Xicheng District, Beijing, 100034 China; 2 Department of Gynecology, The First Central Hospital of Baoding City, Baoding, 071000 Hebei China

**Keywords:** p16^INK4a^ immunostaining, High-grade squamous intraepithelial lesions, Human papillomavirus, Cervical cancer, Colposcopically directed cervical biopsy (CDB)

## Abstract

**Background:**

This study aims to evaluate the value of p16^INK4a^ immunostaining for high-grade squamous intraepithelial lesions in human papillomavirus-negative patients in Beijing, China.

**Methods:**

In this study, we evaluated the value of p16^INK4a^ immunostaining, as well as cytology and colposcopy, for predicting high-grade squamous intraepithelial lesions (HSIL) in human papillomavirus (HPV)-negative patients by comparing the methods with the haematoxylin and eosin (H&E) staining pathological diagnosis of HPV-negative patients.

**Results:**

Of 122 patients negative for the high-risk HPV (hrHPV) subtype, 26 (21.3%) underwent colposcopically directed multiple punch cervical biopsies with H&E pathological diagnoses of HSIL and above (HSIL+), 11 patients (9.0%) had cervical intraepithelial neoplasia (CIN)2, nine patients (7.4%) had CIN3 and six patients (4.9%) had infiltrating carcinomas. Cytology, colposcopy and p16^INK4a^ immunostaining had 52.4%, 38.5% and 92.3% sensitivity, respectively, and 76.2%, 94.8% and 99% specificity, respectively. The positive predictive value of the cytology, colposcopy and p16^INK4a^ immunostaining was 31.4%, 66.7% and 96%, respectively, and the negative predictive value was 88.5%, 85.1% and 97.9%, respectively. Compared with H&E staining, the kappa of the cytology, colposcopy and p16^INK4a^ immunostaining was 0.327, 0.323 and 0.926, respectively.

**Conclusion:**

Positive p16^INK4a^ immunostaining is very strongly consistent with an H&E diagnosis of CIN2+, and it can be used as an objective detection index for HSIL+ diagnoses of HPV-negative patients with CIN2+.

## Introduction

Cervical cancer is the third most common malignancy in women worldwide [1]. Much epidemiological data proves that invasive carcinoma of the cervix is closely related to persistent infection by the high-risk human papillomavirus (hrHPV) subtype [2]. Regular cervical cancer screening for women of reproductive age can reduce morbidity and mortality rates. Cytological screening is used for cervical cancer but faces disadvantages such as low sensitivity and a lack of cytologists. Based on cervical cancer screening of the population in recent years, many studies have found that combined screening with cytology + hrHPV subtype detection and preliminary screening with the hrHPV subtype alone enhances the detection rate [3].

The management of women with negative HPV detection and the identification of an objective index that can reveal the presence of a lesion and the lesion grade is critical. Recent studies have mainly focused on finding alternative biomarkers of cervical cancer. Furthermore, the diagnosis for suspected cervical lesions with negative hrHPV subtype detection and women with invasive carcinomas of the cervix currently depends mainly on the pathological diagnosis, but morphological diagnoses with haematoxylin and eosin (H&E) staining alone is greatly affected by the individual differences. And because the cytological types of cervical lesions are diverse including intraepithelial cell abnormalities, Atypical squamous cells of undetermined significance (ASC-US), Low-grade intraepithelial lesion (LSIL), High-grade squamous intraepithelial lesion (HSIL), Atypical squamous cells can specify HGSIL (ASC-H) and Atypical glandular cells- not squamous carcinoma (AGC-NOS), otherwise, it may lead to misdiagnosis and missed diagnosis of cervical lesions [4]. The evaluations of different pathologists on lesions of the same grade are inconsistent, particularly when diagnosing cervical intraepithelial neoplasia (CIN).

The tumour suppressor protein p16^INK4a^ is a cyclin-dependent kinase (CDK) inhibitor inactivated in many cancers. This inactivation leads to the inactivation of the retinoblastoma protein (Rb). However, in HPV-associated tumours, the HPV E7 protein will combine with Rb and inactivate it. In this process, p16^INK4a^ levels increase markedly [5]. Currently, positive p16^INK4a^ immunostaining can be used as a marker of high-grade squamous intraepithelial lesions (HSIL) [5]. At present, p16^INK4a^ immunostaining is widely used for the precise diagnosis of HPV-related diseases, especially those patients with CIN2 [6, 7]. However, there is insufficient research on p16^INK4a^ in HPV-negative patients. Therefore, we conducted this study to evaluate the value of p16^INK4a^ immunostaining for high-grade squamous intraepithelial lesions in HPV-negative patients in Beijing, China.

## Materials and methods

### Subjects

This was a retrospective study. From January 2014 to December 2014, patients who were negative for the hrHPV subtype were recruited in this study to evaluate the value of p16^INK4a^ immunostaining for detecting HSIL and above (HSIL+). The population included 122 women who were uninfected or previously infected with HPV and previous CIN with or without treatment. These patients had undergone colposcopy and colposcopically directed multiple punch cervical biopsies at our hospital. In our study, even cases with HPV neg/cytology neg underwent colposcopy because these patients had several clinical symptoms of cervical lesions, and the most common was abnormal vaginal bleeding. All biopsy tissues underwent pathological examination. This study was conducted in accordance with the Declaration of Helsinki and approved by the Human Ethics Committee of Peking University First Hospital. All methods were carried out by the WHO guidelines for screening and treatment of cervical pre-cancer lesions for cervical cancer prevention [8]. All eligible participants provided written, informed consent to be included in this study.

### Inclusion and exclusion criteria

Inclusion criteria: (1) patients who were negative for the hrHPV subtype; (2) older than 18 years of age; (3) patients who signed the informed consent. Exclusion criteria: (1) pregnant or nursing women; (2) patients whose data were incomplete.

### Cytological detection

A liquid-based, thin-layer cytologic preparation was used, and the 2001 Bethesda System (TBS) was used for diagnosis [9]. Tissues were evaluated as within normal limits (WNL), ASC-US, ASC-H, LSIL, HSIL, squamous cell carcinoma (SCC) and atypical glandular cells (AGC).

### HPV detection

HPV was tested using the *digene* Hybrid Capture 2 (HC2) High-Risk HPV DNA Test (QIAGEN, Gaithersburg, MD, USA) with the Rapid Capture System (QIAGEN), which is based on signal amplification using RNA probes to target the entire hrHPV genome [10]. All steps were performed according to the manufacturer’s protocols. Briefly, cervical brush samples collected in preserved cytological solution underwent denaturation, hybridisation, capture and amplification of chemiluminescent signal detection. We also used the HybriMax HPV blot (Hybribio Ltd., China), which captures 21 HPV genotypes: namely six low-risk types (HPV 6, 11, 42, 43, 44 and CP8304) and 15 HR types (HPV 16, 18, 31, 33, 35, 39, 45, 51, 52, 53, 56, 58, 59, 66 and 68) that are common in the Chinese population [11]. The HC2 test was used for 43 patients, and the HPV blot was used for 79 patients.

### Colposcopy

Patients with cytological LSIL+ and AGC underwent colposcopy. Patients with no cytological abnormalities or HPV-negative ASC-US but with suspected clinical symptoms (such as contact bleeding, irregular vaginal bleeding, increased vaginal discharge, etc.) of cervical cancer also underwent colposcopy. Colposcopy was carried out per standard procedures. We carried out a colposcopically directed multiple punch cervical biopsy for the most abnormal part of the suspected lesion. The cervical four-quadrant randomised biopsy and endocervical curettage were used when the colposcopy was unsatisfactory.

### Pathological diagnosis of cervical biopsy samples

We used a three-level classification method for the pathological H&E-stained sections of CIN1, CIN2 and CIN3. CIN1 was considered as LSIL; CIN2 and CIN3 were classified as HSIL.

### Detection of p16^INK4a^ protein and evaluation of positive immunostaining results

The immunohistochemical method was adopted for detecting p16^INK4a^. Paraffin sections of cervical tissue were stained according to the reagent kit instructions (Ventana Medical System, Inc., Arizona, USA). We used 1:100 dilution of the primary antibody (mouse anti-human p16^INK4a^ monoclonal antibody; clone number E6H4, USA). The primary antibody was replaced with phosphate buffer solution to construct the negative control; known p16^INK4a^-positive pancreas sections were used as the positive control.

Cells with positive p16^INK4a^ immunostaining had brownish-yellow nuclei and cytoplasm. We determined the staining grade according to the percentage of p16^INK4a^-positive cells: positive, epithelial diffuse layer staining; focal positive, focal, discontinuous positive staining; negative, no obvious staining.

### Cervical loop electrosurgical excision procedure

For patients with H&E pathological diagnosis of CIN2 and above (CIN2+), the cervical loop electrosurgical excision procedure **(**LEEP) was carried out during the next menstrual cycle, and the obtained sample was once again pathologically diagnosed.

### Statistical analysis

We used the software program SPSS 13.0 (SPSS, Chicago, IL, USA) to conduct the statistical analysis. The continuous variables of normal distribution were expressed as mean ± standard deviation, the continuous variables of non-normal distribution were expressed as a median (interquartile range [IQR]), and the categorical variables were expressed as a frequency (percentage [%]). For two comparisons, each value was compared by the *t*-test. For multiple comparisons, each value was compared by one-way ANOVA following a Dunnett’s test when each datum conformed to a normal distribution, while the non-normally distributed continuous data were compared using non-parametric tests. The chi-square test analysed the counting data. A value of *P* < 0.05 was considered statistically significant.

## Results

### The general characteristics

A total of 122 hr HPV-negative patients were included in this study. The age of these participants ranged from 19 to 77 years old. The average age was 42.45 ± 11.33 years. Table 1 lists the details.

**Table 1:** Distribution of patient age

Age (years)	< 25	25–34	35–44	45–54	55–64	≥ 65
Percentage	3.3%	22.1%	35.2%	23.8%	12.3%	3.3%
	(4/122)	(27/122)	(43/122)	(29/122)	(15/122)	(4/122)

### HPV detection results

All of the 122 patients tested negative for HPV (100%).

### Cervical biopsy pathological results

Of the 122 patients, 76 (62.3%) had the pathological result of inflammation, 20 (16.4%) had CIN1, 11 (9.0%) had CIN2, 9 (7.4%) had CIN3 and 6 (4.9%) had SCC. In total, 26 patients (21.3%) were diagnosed with CIN2+.

### Value of cytology for detecting HSIL

We divided the cytology screening results into low-grade abnormalities (WNL, ASC-US, LSIL) and high-grade abnormalities (ASC-H, HSIL, AGC). Of the 26 patients with CIN2+, 12 (46.2%) were in the low-grade abnormality group and 14 (53.8%) were in the high-grade abnormality group. CIN2+ detection between the cytology and cervical H&E pathological results was statistically significantly different (Table 2). The κ value for high-grade abnormalities for the cytology and pathological diagnosis of CIN2+ was 0.327 (χ^2^ = 13.173, *P* = 0.001).

**Table 2:** Comparison of cytology results and cervical biopsy pathological results

Pathological result	Inflammation		CIN1		CIN2		CIN3		SCC		Total	χ^2^	*P*
	n	%	n	%	n	%	n	%	n	%	n		
Cytology results													
WNL	37	78.7	6	12.8	1	2.1	1	2.1	2	4.3	47		
ASC-US	14	66.7	2	9.5	2	9.5	1	4.8	2	9.5	21		
LSIL	10	52.6	6	31.6	2	10.5	1	5.3	0	0.0	19	38.275	0.008
ASC-H	6	46.2	4	30.8	0	0.0	3	23.1	0	0.0	13		
HSIL	5	29.4	2	11.8	5	29.4	3	17.6	2	11.8	17		
AGC	4	80.0	0	0.0	1	20.0	0	0.0	0	0.0	5		

### Value of colposcopy for detecting HSIL

Of the 122 hrHPV-negative patients who underwent colposcopy, 62 (50.8%) were diagnosed as WNL, 45 (36.9%) were LSIL, 11 (9.0%) were HSIL and four (3.3%) were diagnosed with infiltrating carcinomas. We divided the colposcopy results into low-grade abnormalities (WNL and LSIL) and high-grade abnormalities (HSIL and infiltrating carcinoma). Eighteen patients (14.8%) had a high-grade abnormality. There was a statistical difference for CIN2+ detection by colposcopy and H&E pathological results (Table 3). The κ value of the high-grade abnormality for the colposcopy and pathological diagnosis of CIN2+ was 0.323 (χ^2^ = 13.164, *P* = 0.001).

**Table 3:** Comparison of colposcopy results and cervical biopsy pathological results

Pathological results	Inflammation		CIN1		CIN2		CIN3		SCC		Total	χ^2^	*P*
	n	%	n	%	n	%	n	%	n	%	n		
Colposcopy results													
WNL	44	75.9	6	10.3	5	8.6	7	12.1	0	0.0	62		
LSIL	30	66.7	11	24.4	2	4.4	1	2.2	1	2.2	45	70.400	0.000
HSIL	2	18.1	2	18.1	4	36.4	0	0.0	3	27.3	11		
Carcinoma	0	0.0	1	25.0	0	0.0	1	25.0	2	50.0	4		

### Value of p16^INK4a^ immunostaining for detecting HSIL

Of the 122 hrHPV-negative patients that underwent p16^INK4a^ immunostaining, 25 (20.5%) had positive staining, 20 (16.4%) had focal positive staining and 77 (63.1%) had negative staining. We divided the p16^INK4a^ immunostaining results into low-grade abnormalities (negative and focal positive staining) and high-grade abnormalities (positive staining). There was a statistical difference for CIN2+ detection by p16^INK4a^ immunostaining and H&E pathological results (Table 4). The κ of the positive p16^INK4a^ immunostaining and pathological diagnosis of CIN2+ was 0.926 (χ^2^ = 104.59, *P* = 0.000).

**Table 4:** Comparison of p16^INK4a^ immunostaining results and cervical biopsy pathological results

Pathological results	Inflammation		CIN1		CIN2		CIN3		SCC		Total	χ^2^	*P*
	n	%	n	%	n	%	n	%	n	%	n		
p16^INK4a^ immunostaining results													
Negative	69	89.6	8	10.4	0	0.0	0	0.0	0	0.0	77		
Focal positive	7	35.0	11	55.0	2	10.0	0	0.0	0	0.0	20	134.760	0.000
Positive	0	0.0	1	4.0	9	16.0	9	36.0	6	24.0	25		

### Lower anogenital squamous terminology project HSIL diagnosis of cervical biopsy tissue

**Fig. 1:**
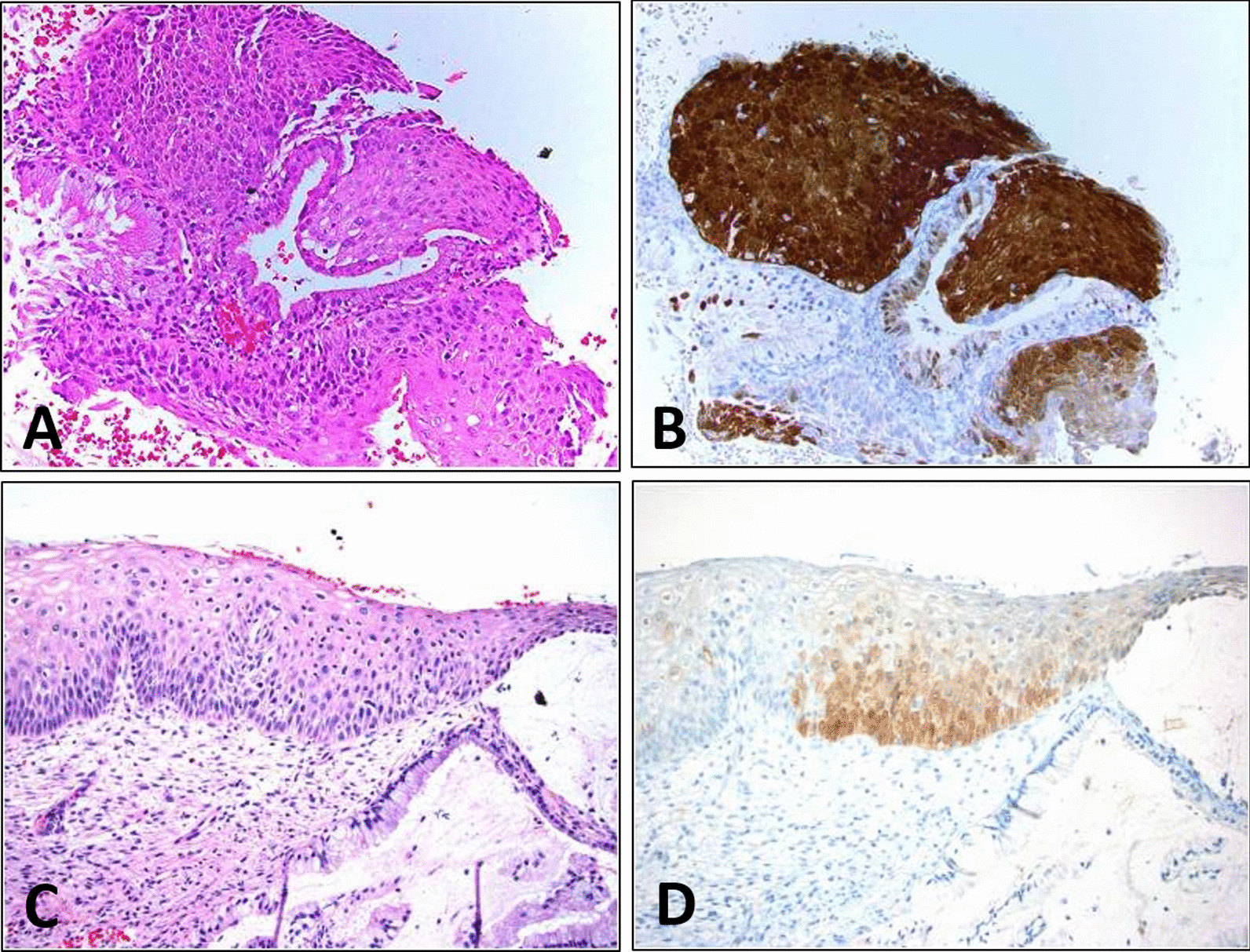
The images of typical positive cases.** A** is the HSIL of HE staining,** B** is the corresponding P16 staining;** C** is the LSIL of HE staining,** D **is the corresponding P16 staining. The P16 of HSIL is deeply stained, with large brown areas. LSIL's P16 stain is light and flaky yellow

In lower anogenital squamous terminology **(**LAST), the diagnostic terminology for different parts of the anus and reproductive tract and the pathological diagnosis of cervical tissue is consistent with TBS terminology [12–14]. In this study, classification based on the cytology, colposcopy and H&E pathology detected 26 cases of HSIL+ (CIN2+) (Fig. 1); based on LAST, and there were 24 cases of HSIL+. The differences were the two cases that were focally positive for p16^INK4a^. After cervical LEEP, one case was diagnosed as CIN1, i.e. LSIL, another was diagnosed as CIN2, i.e. HSIL. The two high-grade lesions were not considered in the LAST.

### Pathological results of CIN2+ after cervical LEEP

All 26 patients with H&E pathological diagnoses of CIN2+ underwent cervical LEEP, of which 24 had positive p16^INK4a^ immunostaining, and two had focal positive p16^INK4a^ immunostaining. The latter two patients underwent cervical LEEP and returned the pathological results of CIN1 and CIN2, respectively. Of the 122 patients included in the study, 25 were p16^INK4a^-positive: one patient with punch biopsy findings of CIN1 is currently under follow-up; the remaining 24 underwent cervical LEEP, and the LEEP specimen pathological results were CIN1 (three patients), CIN2 (nine), CIN3 (six) and infiltrating carcinoma (six patients). There was a statistically significant difference between the punch biopsy pathological results under colposcopy and after cervical LEEP (χ^2^ = 31.704, *P* = 0.000) (Table 5). The positive predictive value (PPV) of positive p16^INK4a^ expression for the pathological HSIL+ after cervical LEEP was 87.5%.

**Table 5:** Comparison of the pathological results before and after cervical LEEP in p16^INK4a^-positive patients

Colposcopically directed cervical biopsy pathological results	Pathological results after cervical LEEP								
	CIN1		CIN2		CIN3		Infiltrating carcinoma		Total
	n	%	n	%	n	%	n	%	
CIN2	3	33.3	5	55.6	1	11.1	0	0.0	9
CIN3	0	0.0	4	44.4	5	55.6	0	0.0	9
Infiltrating carcinoma	0	0.0	0	0.0	0	0.0	6	100.0	6
Total	3	12.5	9	37.5	6	25.0	6	25.0	24

### Comparison of detection methods for cervical precancerous lesions

In summary, H&E staining is the gold standard for detecting cervical precancerous lesions. We evaluated the cytology, colposcopy and p16^INK4a^ immunostaining for detecting cervical HSIL in cervical HPV-negative patients (Table 6). The sensitivity, specificity, PPV and negative predictive value (NPV) of p16^INK4a^ immunostaining were all > 90%, significantly better than cytology and colposcopy. Including focally positive p16^INK4a^ immunostaining in the detection of cervical precancerous lesions increased the sensitivity and NPV to 100% but greatly reduced the specificity and PPV, meaning it is not the best choice.

**Table 6:** Comparison of different methods for detecting cervical precancerous lesions

Parameter	Cytology	Colposcopy	p16^INK4a^ immunostaining (positive staining)	p16^INK4a^ immunostaining (positive and focal positive staining)
Sensitivity	52.4	38.5	92.3	100
Specificity	76.2	94.8	99.0	80.2
PPV	31.4	66.7	96.0	57.8
NPV	88.5	85.1	97.9	100
Kappa with H&E staining	0.327	0.323	0.926	

## Discussion

Numerous epidemiologic studies have proven that persistent hrHPV infection is the main causative factor of HSIL and infiltrating carcinoma [3]. hrHPV infection can be detected in almost all patients with precancerous lesions and invasive carcinomas of the cervix, and there would be positive clinical detection of an hrHPV subtype. However, a certain proportion of patients with precancerous lesions and invasive carcinomas of the cervix may be negative for hrHPV testing, and the management of such patients should be investigated in particular. At present, hrHPV detection enhances screening sensitivity when used as an objective index for cervical cancer screening (early or combined cytological screening) [2, 15, 16]. However, there is currently a lack of more sensitive and objective screening diagnosis indexes for hrHPV-negative women.

Previous studies have found that the reproducibility of H&E staining morphological diagnoses by pathologists is poor and that the consistency of the CIN2 diagnosis is < 50% [17]. The reproducibility of CIN2+ morphological diagnosis in the ASC-US/LSIL Triage Study for Cervical Cancer (ALTS) was only 43% [18]. In another study, two pathologists agreed with 84% and 81%, respectively, for the CIN3 diagnosis; for CIN2, the agreement was 13% and 31%, respectively [19]. There are vast differences among pathologists for H&E staining diagnoses of CIN2, and there are many false-positive or false-negative results. Recent studies have suggested that the auxiliary use of immunohistochemical staining may aid the accuracy of the CIN2 diagnosis.

The p16^INK4a^ protein can compete with cyclin D1 to bind with CDK4, inhibiting CDK4 activity. The p16^INK4a^/CDK-cyclin D/Rb complex is the key factor when cells exit the G1 phase of the cell cycle and enter the S phase. Any gene abnormality in this molecular chain may result in the loss of control of molecular action on the cell cycle. The E7 protein expressed by hrHPV interferes with the normal function of the *RB* gene. E7 binds with phosphorylated Rb (pRb), inactivating the function of the *RB* gene, eliminating the negative feedback inhibition of pRb on p16^INK4a^ protein expression, resulting in p16^INK4a^ overexpression [4]. This leads to a disorder of the cell cycle of the cervical epithelial cells, resulting in the characteristic of immortality and the initiation of a series of carcinogenesis processes [20–23].

Li et al. from our department carried out p16^INK4a^ and Ki-67 immunostaining on the pathological sections of patients with CIN aged < 35 years. These researchers found that p16^INK4a^ and Ki-67 immunostaining had very good consistency with CIN grading [24]. Galgano et al. found that sensitivity was 86.7% and specificity was 82.8% for CIN2+ and that p16^INK4a^ immunostaining is a useful and reliable diagnostic adjunct for distinguishing biopsies with and without CIN2+ [25]. Bergeron et al. found that p16^INK4a^-immunostained slides significantly increased the diagnostic accuracy for detecting high-grade CIN compared with H&E slides and that the reproducibility of p16^INK4a^ immunostaining interpretation was excellent [26]. Other investigators suggested that the conjunctive use of H&E morphology with p16^INK4a^ immunostaining improved the inter-observer agreement of the CIN2+ diagnosis [27]. The LAST Project working group of the College of American Pathologists and ASCCP proposed that the addition of p16^INK4a^ immunostaining in some cases may provide a more reliable and consistent pathological interpretation [12, 13]. However, can p16^INK4a^ be used as a molecular biological substitute for pathological HSIL of the cervical tissues in hrHPV-negative patients? Zhang et al. found that diffuse p16 immunostaining is the hallmark of HSIL because it correlates 100% with CIN2 and CIN3 lesions between the initial biopsy and cervical LEEP specimens, whatever the HPV status [28]. Solano et al. found that p16^INK4a^ immunostaining had more diagnostic benefits, where their retrospective study of 596 patients revealed HSIL/CIN2–3 was not found in the initial H&E staining [29].

There was a very strong consistency between positive p16^INK4a^ immunostaining and H&E staining pathological diagnoses in our study (κ = 0.926). The PPV and NPV of high-grade lesion diagnosis were 92.3% and 97.9%, respectively. Almost 100% of the cervical HSIL or infiltrating carcinoma could be excluded for patients with negative p16^INK4a^ immunostaining. For the follow-up of the pathological results, we found that the PPV of p16^INK4a^-positive staining for pathological HSIL+ after cervical LEEP was 87.5%, which is higher than that reported by Clinton et al., who found that HSIL detection increased from 48 to 76% (*P* < 0.05) after the wide clinical application of p16^INK4a^ immunostaining [13]. Besides, in our study, two patients with H&E-diagnosed CIN2 had focal positive p16^INK4a^ immunostaining. The diagnoses of these two patients would be classified as LSIL according to the recommendations of the 2012 LAST guidelines, and follow-up may be conducted for management. The two patients also underwent cervical LEEP in the next menstrual cycle after cervical biopsy, and the results were CIN1 and CIN2, respectively.

Immunostaining for p16^INK4a^ can be used as a molecular biological substitute for evaluating pathological HSIL of cervical tissue in hrHPV-negative patients and can be used to aid HSIL detection.

## Limitations of the study

This study had some limitations. Firstly, this study was a single-centre trial, so the sample size was limited. Multiple centre trials with a large sample size are still needed in the future. Secondly, diagnostic cervical LEEP can be conducted for older women, women with persistent CIN2 for > 2 years or patients with other risk factors. As there were only two such patients in this study, more cases should be gathered in the future for an in-depth study to facilitate suggestions for suitable management. Lastly, it will be better for study purposes to have two hrHPV tests to confirm the possibility of an hrHPV-negative HSIL. Test failures, low dosage of HPV or potential carcinogenic types may elude detection by just one test. Besides, although the included samples were HPV negative, the methods cannot rule out infection with a genotype not included within the assays used.

## Data Availability

All data generated or analyzed during this study are included in this published article.

## Abbreviations

HSIL: High-grade squamous intraepithelial lesions
HPV: Human papillomavirus
H&E: Haematoxylin and eosin
HR-HPV: High-risk HPV
CIN: Cervical intraepithelial neoplasia
CDB: Colposcopically directed cervical biopsy
H&E: Haematoxylin and eosin
CDK: Cyclin-dependent kinase
HSIL: High-grade intraepithelial lesions
TBS: Bethesda system
WNL: Within normal limits
ASC-US: Atypical squamous cells of undetermined significance
ASC-H: Atypical squamous cells—cannot exclude high
LSIL: Low-grade squamous intraepithelial lesion
SCC: Squamous cell carcinoma
AGC: Atypical glandular cells
HC2: Hybrid capture 2
PBS: Phosphate buffer solution
LEEP: Loop electrosurgical excision procedure
IQR: Interquartile range
ASCCP: American Society for Colposcopy and Cervical Pathology
PPV: Positive predictive value
